# Clinical Cases

**Published:** 1892-01

**Authors:** W. E. Ledyard

**Affiliations:** San Francisco, Cal.; Clinical Bureau, I. H. A.


### CLINICAL CASES.

W. E. Ledyard, M. D., San Francisco, Cal.

(Clinical Bureau, I. H. A.)

Case No. 1.—December 22d, 1890. Woman, plump and fair, aged nineteen; one month married; complained of severe cutting and pressing pains, worse by pressure, in left ovarian region. Leucorrhœa, constant, yellow, thick or thin. Last night took Dover's powder and hot drinks. Gave *Belladonna*$^{200}$ every half-hour in solution until relieved. A few doses gave complete relief.

Case No. 2.—Whooping Cough.—February 11th, 1891. Male child, aged five months, plump, fair. Analysis of symptoms as follows:

1. Crying during paroxysm of cough: Ant-t., Arn., Ars., *Bell.*, Cham., Chin., *Cina*, Hep., Ip., Lyc., Sep., Sil., Samb., Verat.
2. Wakened by the cough: Bell., Hep., Sep., Sil.
3. Cough, with red face: Bell., Hep., Sil.
4. Cough, lachrymation: Hep., Sil.
5. Cough, salivation: Hep.
6. Cough, watery nasal discharge, appeared to call for remedies not in the above list. One dose of *Hepar.*$^{200}$ was given, followed by Placebo.

February 12th.—Cough, during the night, somewhat more frequent. Continued Placebo.

February 13th.—Cough about the same—is accompanied by *rattling.*

*Hepar*$^{200}$, dose at once, and again at three P. M. next day. Continued *Placebo.*

February 16th, 17th, and 18th.—Hepar$^{200}$ was given night and morning. On the 16th the cough was considerably more frequent, especially after midnight; on 17th, cough more frequent from half-past ten P. M. to two A. M.

February 19th.—Cough not so frequent nor so severe.

*Hepar*$^{200}$, night.

February 21st.—Cough again worse after midnight.

*Hepar*$^{200}$, night and morning.

February 23d.—1. Crying during the cough: Ant-t., Arn., Ars., Bell., Cham., Chin., *Cina.*, HEP., Ip., Lyc., Sep., Sil., Samb., Verat.

2. Wakened by the cough: Bell., Hep., Sep., Sil.

3. Red face during cough: Bell., Hep., Sil.

4. Coryza during cough: Bell., Sil.

5. Stiffness and rigidity of body during cough: Bell.

Belladonna$^{200}$ was given night and morning until March 2d, when, there being a decided improvement, the Belladonna$^{200}$ was given only at night until, March 5th, still further improvement showing itself, the Belladonna was given every other night, and the cough became less and less frequent, and in a few days disappeared.

The case was under treatment twenty-two days.

CASE No. 3.—PNEUMONIA.—February 16th, 1891. Male child, aged two and one-half years, fair complexion, large head, very cross; objects to being looked at, spoken to, or touched.

At night, face red with thirst; waking up frightened.

Eyes dull; breathing hurried; cough—short, dry, with sweat on forehead. Auscultation showed crepitant râles at base of right lung, posteriorly.

Ant-t.$^{200}$, dose, repeat night and morning, if necessary. A few doses cured.

CASE No. 4.—PNEUMONIA.—Was called on June 2d, 1891, to see a boy, aged ten years and ten months old, of a leucophlegmatic constitution, with the following history: May 31st, appetite ravenous. June 1st, at six A. M., retching, followed by vomiting of a little green mucus.

In the evening was much worse; skin hot and very dry; *lips* and mouth *dry;* about midnight became *very thirsty* and called for *ice.*

June 2d.—*Nausea, worse on raising the head;* delirium; talking of things sticking in throat; wing-like motion of alæ nasi; moaning; cheeks deeply flushed; cough loose and hacking; bowels inclined to be costive; expectoration *rust-colored; doesn't*

*wish to be touched or moved.* Pulse, 136. Half-past five P. M., *Bryonia*$^{200}$, in solution, every half-hour, until relieved.

June 3d.—Improvement set in nine P. M., yesterday. Half-past eleven A. M., no *Bryonia* since nine P. M., yesterday. Complains of pain in head and pit of stomach. Gave one dose of *Bryonia*$^{200}$. Half-past five P. M., headache distressing. *Pulse*, 120. *Bryonia*$^{200}$, in solution, every half-hour for three doses.

June 4th.—So much better that I found him awaiting my visit with smiling face, and sitting up after a good night's rest. Pulse, 100. Placebo.

June 5th.—The improvement continues, a slight cough only remaining. *Bryonia*$^{1000}$, dose.

June 6th.—Cough about the same. *Bryonia*$^{40m}$, dose.

June 17th.—His mother called at the office to consult me about two other children, accordingly I pronounced above patient well. The above case was under treatment six days, and the pulse dropped from 136 to 100 in forty-eight hours.

CASE No. 5.—TONSILLITIS.—Girl, aged eleven, brown eyes, stout. Throat very sore; worse when swallowing solid food, the pain extending to the right ear; tonsils swollen; very red; tenderness of neck; greenish mucus adheres to posterior wall of pharynx, and it is impossible to expectorate, exciting cough.

Apis and Kali-bichromicum both have "pain extending to the ears when swallowing" and tenderness of the neck, but the pain was greater and the swelling less than we find in the Apis case, and these facts, with the presence of the *tenacious* mucus, decided in favor of *Kali-bichromicum*, which was given *night and morning* in the 200th potency, and *in twenty-four hours* the improvement was very great and the mucus had almost disappeared, and the patient was continued on a *Placebo*.

CASE No. 6.—Woman, aged thirty; light complexion; leucophlegmatic; pregnant five months.

Before marriage was subject to coldness and numbness of the hands at night.

Present symptoms for two or three weeks:

*Hands:* sensation in daytime as if half dead, stiff and scalded; at night, heat and burning, worse in the right, with swelling and bursting and severe aching pains, which woke her at two A. M., lasting two hours. The pains extend to the axilla, being always worse on the right side. Sometimes is compelled to rise from bed and walk the floor for relief.

Micturition frequent, with painful bearing down in uterine region and burning in the urethra.

Has applied turpentine to the hands.

After being under my treatment for about a month without relief, she informed me that a short time before the present symptoms came on, she had been *washing lime from the walls and floor*. The hands are *worse* after putting them into hot or cold water ; also at night ; while ironing (from the heat of the iron) ; and *much worse from letting the hands hang down*.

She now received one dose of *Calc-c.*$^{200}$.

A week after there was a decided improvement, but toothache, with alveolar abscess in right upper jaw, with exquisite soreness, pain extending to eyes and ears and sore throat, worse right side, imperatively demanded Belladonna, which was given in the 200th potency, in solution, every half-hour, until relieved.

The following week reported that the abscess discharged on the way home, and the pain speedily disappeared. The hands are improving. One dose of *Calc-c.*$^{500}$.

Another week passed, and I had a letter from the patient's husband as follows :

" My wife desires me to say that she has so far recovered as to be able to get along without further medical aid. Thanking you for the interest you have taken in her case," etc.

Three weeks later the patient was still improving, the hands being all right.

CASE NO. 7.—November 4th, 1879.—Woman, aged, forty, single, tall, of dark complexion, seven weeks ago, while carrying a child in the arms, *fell on the left knee*.

For ten days she has had a large fluctuating swelling over the left patella, with pains in the limb.

Sticta-pulmonaria$^{30}$ night and morning.

November 15th.—The pains have disappeared ; otherwise the symptoms remain as before.

Sticta-pulmonaria$^{200}$ at night for a week.

November 25th.—Patient reports that the swelling began to decrease on the 19th, and by the 21st had altogether disappeared.

In Hering's *Condensed Materia Medica* we read, under Sticta-pulmonaria, "Bursitis, especially about the knee."

She afterward informed me that several well-known allopathic surgeons had proposed to "operate" on the knee. Old-school authorities recommend "rest," with or without "cooling" lotions. In more chronic cases, *pressure* with large sponge, wetted and bound on tightly with a roller, *and rest;* blisters; the external application of tincture of iodine; long open incisions, followed by *complete rest* for one or two weeks, *and* warm *fomentations* or *poultices*. In case the sac doesn't granulate and close up, injections of tincture of iodine, carbolic acid, or other stimulants. For large and old bursæ, excision.

